# Supplementary figures and images for: Phosphoproteomic identification of Mos–MAPK targets in meiotic cell cycle and asymmetric oocyte divisions
Source: J Cell Biol. 2025 Oct 22;224(12):e202312140. doi: 10.1083/jcb.202312140 (PMC12542822; doi:10.1083/jcb.202312140)

Fig. 1A

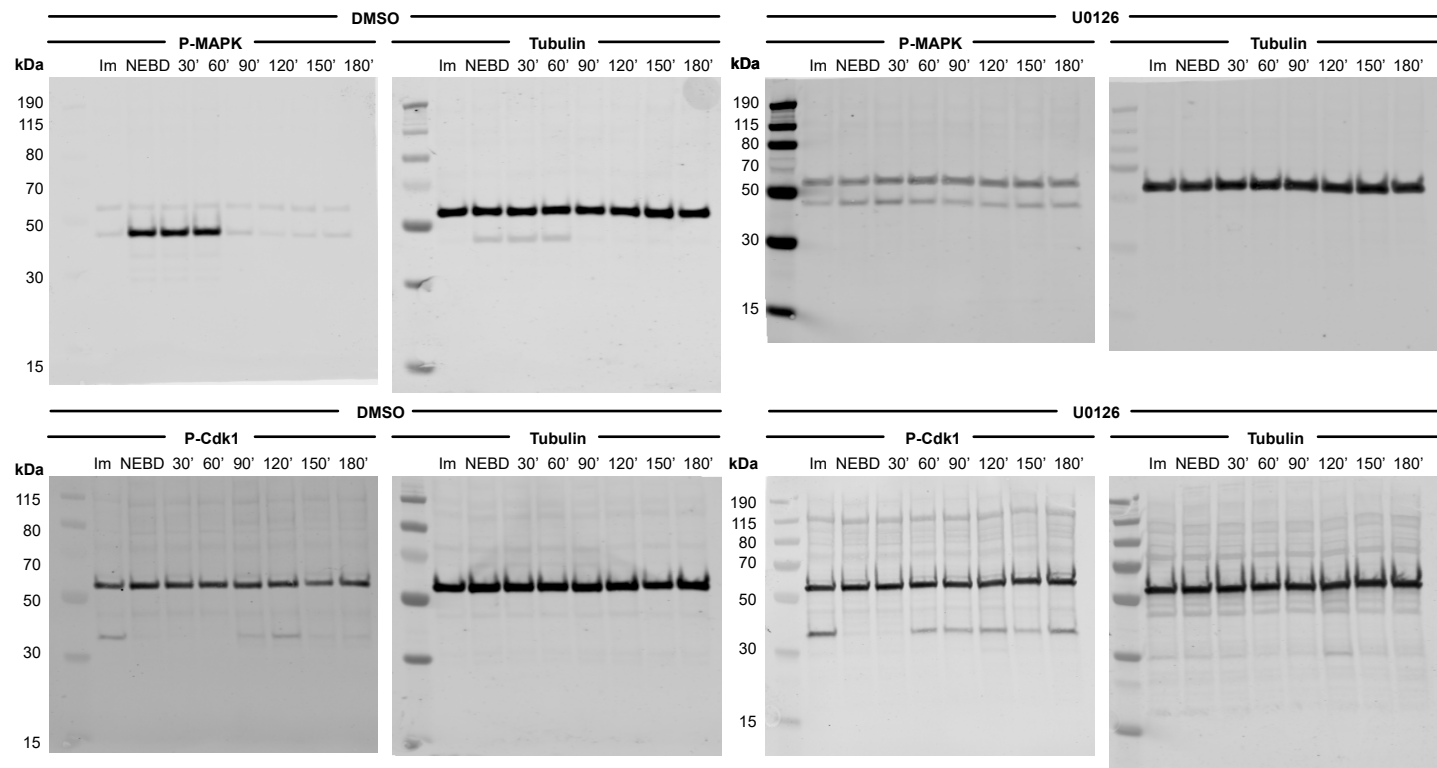

Supplement: SourceData F1 — is the source file for Fig. 1. [file jcb_202312140_sourcedataf1.pdf]

**Fig. S1B**

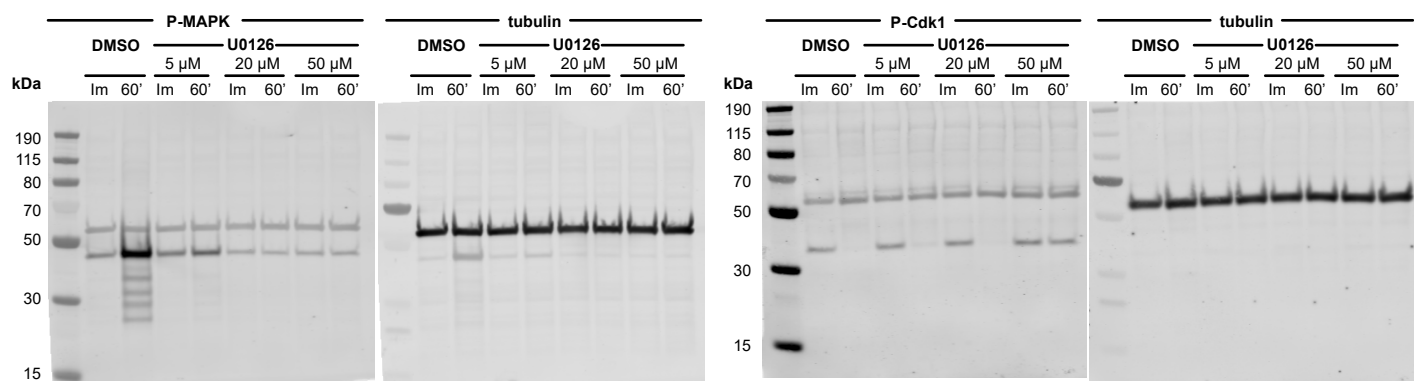

Supplement: SourceData FS1 — is the source file for Fig. S1. [file jcb_202312140_sourcedatafs1.pdf]
